# A Six-Year Hydration Evaluation of Cs-Bearing Materials at Room Temperature and 55% Relative Humidity Simulating Radioactive Waste with Different Crystallinities

**DOI:** 10.3390/molecules29061302

**Published:** 2024-03-14

**Authors:** Guido Cerri, Antonio Brundu

**Affiliations:** Department of Architecture, Design and Urban Planning—GeoMaterials Lab, Sassari University, Via Piandanna 4, 07100 Sassari, Italy

**Keywords:** vapor hydration, amorphous materials, cesium immobilization, CAS-type zeolite, CsAlSi_2_O_6_

## Abstract

Radioactive wastes often contain amorphous and crystalline phases, and vapor hydration can affect their durability. In this study, Cs-clinoptilolite was heated (at 1100 °C and for 2–36 h) to prepare the samples that were composed mainly of an amorphous phase (AmP) and CsAlSi_5_O_12_ (≥94%) with minor CsAlSi_2_O_6_. Six samples with an AmP/CsAlSi_5_O_12_ ratio from 26.5 to 0.1 were kept at 21 °C and 55% relative humidity, and their hydration was measured via thermogravimetry (TG) over a period of almost six years. The hydration that resulted was directly related to the AmP quantity. The increase in water content followed a logarithmic trend over time. It reached 1.95% in the AmP-richest material, while it attained only 0.07% in the most crystalline sample. The hydrolysis of the AmP led to an increase over time in the tightly bound water. Samples with an AmP of ≤19% demonstrated slightly higher durability due to the lower Cs content in the AmP.

## 1. Introduction

^137^Cs is a byproduct of fission processes in nuclear reactors, and it is considered one of the most dangerous radionuclides [1,2]. The controlled releases that occur from operation at nuclear facilities and the nuclear accidents that take place in power plants are among the main sources of ^137^Cs. Ion exchange is an established method for treating contaminated water [3]. In this application, framework aluminosilicates such as certain natural zeolites (e.g., clinoptilolite, chabazite, and mordenite) offer advantages due to their cation exchange capacity, their selectivity toward Cs^+^ and Sr^2+^, their resistance to degradation when exposed to radiation, their low cost, and their wide availability [4,5,6,7]. Clinoptilolite has been used in decontamination operations after the Chernobyl and Fukushima accidents [8,9], and is or has been employed for nuclear waste treatment in at least eleven countries [5], including the Sellafield nuclear reprocessing site (UK) [10]. Spent ion exchangers must be immobilized in a solid waste form to ensure safe disposal, as well as to prevent the release of radioisotopes such as ^137^Cs. Vitrification by heat treatment is a widely used technique to achieve this goal as glassy products have high chemical durability [11]. Zeolites allow one to overcome the problem of cesium volatilization that is typical of conventional vitrification (which requires high-temperature melting [12,13]). However, the amorphization of clinoptilolite in Cs-form can be attained at 1000–1100 °C, and it also produces a material that exhibits a low cesium release [14,15]. On the other hand, by simple heating or through hot-pressing, Cs-clinoptilolite can be transformed into crystalline CsAlSi_5_O_12_(CAS) [16,17,18,19], but it is also possible to favor the nucleation of CsAlSi_2_O_6_ (POL) over CAS [20]. Both phases show features that are suitable for safely hosting ^137^Cs [21,22]. Most importantly, leaching tests have highlighted the higher cesium retention capacity of CAS with respect to its amorphous counterpart [23]. As for nuclear waste immobilization, there is growing interest in glass composite materials, which consist of both amorphous and crystalline phases. In these composites, most of the radioactive elements are locked into the more durable crystals that are, in turn, encapsulated in the glassy matrix [11,13,24]. Radioactive waste is often constituted of heterogeneous products that contain amorphous and crystalline phases, where the components with lower chemical durability are more susceptible to alteration [24,25]. In this perspective, it should be noted that Cs-bearing glasses are highly hygroscopic [26], and this may affect their long-term chemical stability because H_2_O can attack the glass by (i) leaching cations via ion exchange with H^+^/H_3_O^+^ and (ii) depolymerizing the glass network through a hydrolysis led by OH^−^ [25]. Regarding Cs-clinoptilolite, calorimetric measurements have shown that its amorphization produces a material with a significantly higher hydration reactivity [15]. In addition, certain crystalline Cs-aluminosilicates are hygroscopic [27]. However, with respect to CAS, there are no data concerning a possible long-term hydration. The issue of glass alteration in unsaturated atmosphere conditions, i.e., with a relative humidity (RH) < 100%, deserves attention because the material is expected to be first hydrated in water vapor prior to liquid alteration during the geological disposal of radioactive waste, and this hydration process may be detrimental to its durability in subsequent aqueous solutions as pre-vapor-corroded glass is more leachable compared to its pristine counterparts [25,28]. Although the reactions between glass and water are essentially the same for atmospheric and liquid alteration, the kinetics and the products are distinct because of the minimal amount of H_2_O in the vapor phase and the almost no leaching by solution [29]. In a geological disposal site, the vapor hydration of nuclear waste can last for a particularly long time and may represent a significant corrosion process [30]. Hydration is the first step in glass alteration, and an amorphous hydrated layer is the only ever-present alteration product [28,29,30,31,32,33,34]. However, most of the vapor hydration experiments have been performed in extreme conditions (100–200 °C and 100% RH) that may differ from those expected in a geological repository [29], and certain authors have warned about the possible differences in vapor hydration mechanisms at low (35–90 °C) and high (90–200 °C) temperatures [31]. A general mechanism of vapor hydration over time has not yet been established [31]. There is a need to carry out experiments, especially in atmospheric conditions, due to the low kinetics of alteration processes at ambient temperatures [32]. Furthermore, the behavior of a composite waste constituted of amorphous and crystalline phases deserves study. The present work aims to provide a contribution to deepening the understanding of these themes. Starting from a previously prepared Cs-clinoptilolite (Cs-C), a set of samples with the same chemical composition but different contents of amorphous phase (AmP) and CAS were produced by heating Cs-C at 1100 °C at different times (2–36 h). The hydration of the samples, which were stored in atmospheric conditions at approximately 21 °C and 55% RH, was evaluated for almost six years via thermogravimetric (TG) and derivative thermogravimetric (DTG) analyses. Moreover, the hydration over time of the samples subjected to thermal analysis was then compared with the path of hydration followed by the same samples before the analysis.

This study revealed that the hydration of the materials occurred even at room temperature and 55% RH, and that the hydration over time was always directly related to the quantity of the AmP in the samples (with the correlation coefficients *R^2^* ranging from 0.94 to 0.99). The water content reached 1.95% in the AmP-richest material and 0.07% in the poorest one. Logarithmic curves (*R^2^* 0.89–0.99) were used to define the rise in H_2_O content over time for all the samples with AmP ≥ 19%. Hydrolysis of the AmP led to an increase over time of the tightly bound water, which was demonstrated by the shift (≈45 °C) toward higher temperatures of the DTG peak of dehydration. Once subjected to TG analysis, the hydration path of the samples did not trace the initial one but—following a parallel curve—it instead stood slightly below: a phenomenon that occurs due to the re-polymerization of the AmP.

## 2. Results

### 2.1. Composition, Density, and Specific Surface Areas of the Prepared Materials

**Figure 1:**
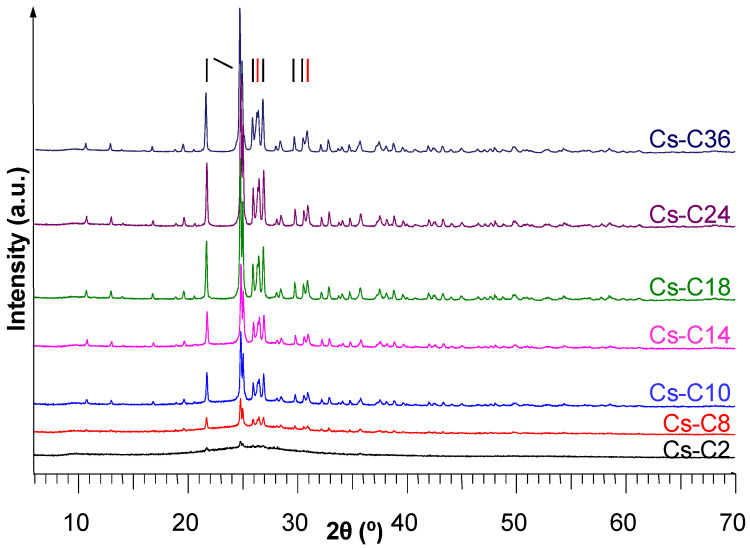
X-ray diffraction patterns of the samples after heating at 1100 °C. The black bars represent CsAlSi_5_O_12_ (CAS) PDF file N° 83-1314 and the red bars represent CsAlSi_2_O_6_ (POL) PDF file N° 29-0407. Only the main peaks of the two phases are labeled here (the complete sets are in Figure S1a–g) (Supplementary Materials).

The X-ray diffraction (XRD) patterns of the samples, which were labeled according to the heating hours spent at 1100 °C (respectively, Cs-C2, Cs-C8, Cs-C10, Cs-C14, Cs-C18, Cs-C24, and Cs-C36), are reported in Figure 1. It clearly shows the progressive crystallization of CAS and subordinate POL from the almost completely amorphous Cs-C2 to Cs-C36.

The transformation of Cs-C, which proceeded according to reactions described elsewhere [19], produces two open framework compounds that—despite their microporous structure—are regarded as ceramic materials [22,23]. CsAlSi_5_O_12_ is the type material of the CAS zeolite topology, whereas POL shows a pollucite-like structure (i.e., the ANA framework type) [23].

**Figure 2:**
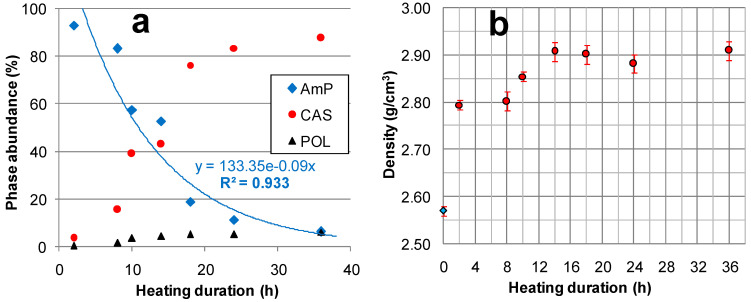
The effect of the heating duration at 1100 °C on (**a**) the phase abundance (in wt%) and (**b**) the density (the density of the unheated powder from ref. [23]).

The results of the XRD quantitative determinations (see Figure S1a–g) are summarized in Table 1. All of the samples were mainly composed of the AmP and CAS (where their sums varied from 93.9 to 98.6%), and the ratio between these phases decreased progressively from 26.5 (Cs-C2) to 0.1 (Cs-C24 and Cs-C36). The quantity of the AmP decreased exponentially with the duration of heating, as can be seen in Figure 2a. A residue of feldspar (3.2%, Table 1) was detectable only in the material heated for 2 h, whereas POL was present in all of the samples in increasing amounts (from 0.4 to 6.1%) alongside the duration of the thermal treatment.

Table 1 also reports the Cs-occupancy factors in the CAS that were determined through the Rietveld refinement. The values are between 0.91 and 0.93 with the sole exception of Cs-C2 (but less confidence should be attributed to its Cs-occupancy due to the low amount of CAS).

Cesium-deficient CAS structures are quite common, and the factors obtained in this study were consistent with the literature data [19,35]. In general, the crystalline phases obtained from clinoptilolite through solid-state transformations have often revealed compositions that deviate from the ideal formulae [36,37]. Due to the scarce quantity of POL in the materials, the refinement of its Cs-occupancy was not allowed during the quantitative determinations (a value of 1 was set, i.e., stoichiometric composition). Having said this, in our experience, POL that is nucleated from Cs-clinoptilolite also shows a defect in cesium [20].

By taking into account (a) the chemical composition of Cs-C on anhydrous base, (b) the amount of CAS and POL formed, and (c) the Cs-occupancy factors of CAS, it is possible to estimate the amount of cesium hosted in the crystalline phases, as well as to obtain—via the difference—the quantity left in the AmP. The data in Table 1 display how the element was distributed among the Cs-bearing phases (see the percentage of Cs_phase_/Cs_sample_), as well as show that, for the longest treatment, the cesium was completely confined in the crystalline compounds. Table 1 also highlights the variation in the AmP composition in terms of the Cs_2_O content, which progressively decreased from 26.9% to zero. In reality, when bearing in mind that the formed POL could also be Cs-deficient, the AmP resulting from the longest treatment should not be completely cesium-free; nevertheless, the described trend remains valid. In other words, not only does the amount of AmP decrease with heating due to CAS and POL crystallization, but the resulting residual AmP is progressively depleted in cesium.

The effect on the density of the duration of heating at 1100 °C is represented in Figure 2b. The density increased by about 10% after a treatment of 2 h, from 2.57 ± 0.01 (unheated Cs-clinoptilolite-bearing powder [23]) to 2.79 ± 0.01 g/cm^3^. Afterward, the density value slowly increased, reaching 2.90 ± 0.02 g/cm^3^ after 14 h of heating, and—within the experimental error—it did not vary again until 36 h.

The specific surface areas (S_BET_) of the prepared powders—which are listed from Cs-C2 to Cs-C36—are 1.17 ± 0.02; 1.41 ± 0.03; 1.78 ± 0.04; 2.31 ± 0.09; 1.80 ± 0.05; 1.19 ± 0.02; and 1.83 ± 0.09 m^2^/g. These values are characteristic of non-porous materials, as stated by Park et al. [38] when commenting on a similar surface area (2.37 m^2^/g), which was referred to a zeolite 13X that had been exchanged with Cs and treated at 900 °C for 2 h. There was no correlation found between the heating duration and S_BET_, and the slight difference in the surface area of the powders could have simply resulted from the micronization process (see Section 4.2). On the other hand, considering the particles as perfect spheres with mean diameters between 1 and 2 μm, and taking into account the measured densities, the calculated surface areas would range from 1.03 to 2.15 m^2^/g. These values were consistent with those observed, as well as with the literature data (Alloteau et al. [34] measured an S_BET_ of 0.78 ± 0.09 m^2^/g for a glass powder of 5–10 μm).

### 2.2. Hydration over Time of the Prepared Materials

Table 2 reports, for each sample, the loss on ignition (LoI) recorded during the thermal analyses. Due to damage to the vial, Cs-C36 was lost during laboratory operations and only one TG analysis was performed on it.

**Figure 3:**
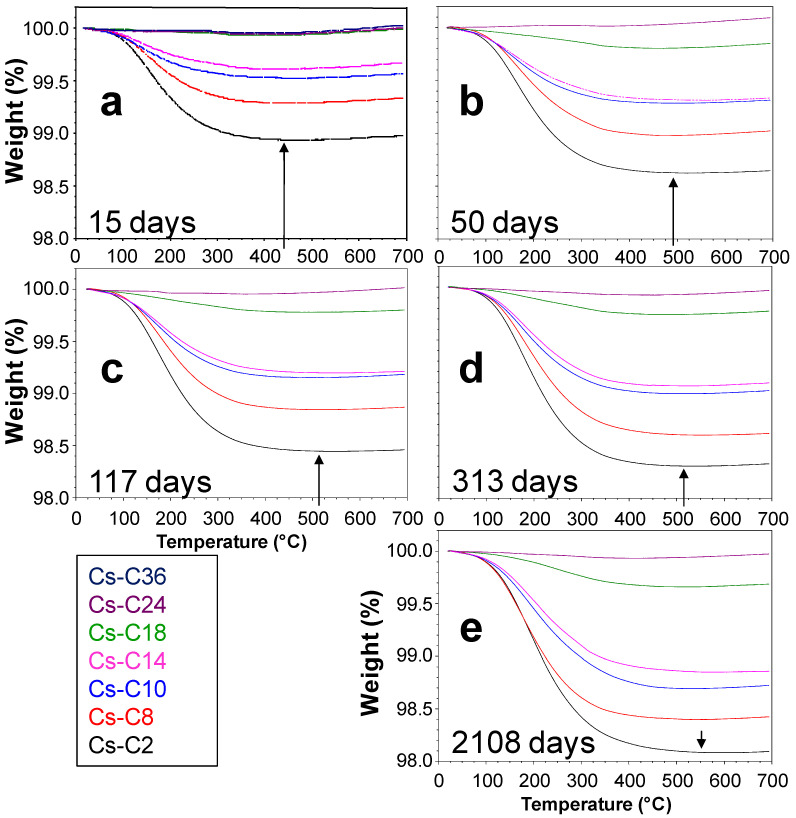
Thermogravimetric (TG) analysis of the materials after (**a**) 15, (**b**) 50, (**c**) 117, (**d**) 313, and (**e**) 2108 days of aging at 21 °C and 55% relative humidity (RH).

The data demonstrated that a detectable hydration of the materials occurred even at room temperature and 55% RH, and that the water content increased over time. Furthermore, with respect to the quantity of H_2_O lost, the samples invariably followed the same order (i.e., Cs-C2 > Cs-C8 > …), as can be seen from the TG curves in Figure 3a–e that refer to the analyses performed 15, 50, 117, 313, and 2108 days after the preparation of the materials.

Generally, the weight loss of the hydrated glass is completed at about 500 °C [32,34]. Our data showed that the time elapsed at the controlled temperature and RH% affected the temperature at which the dehydration ended. For example, the TG curve of the most hydrated material (Cs-C2) clearly showed that the weight loss ended at ≈440 and at ≈550 °C for the aging times of 15 and 2108 days, respectively (see arrows in Figure 3a,e).

**Figure 4:**
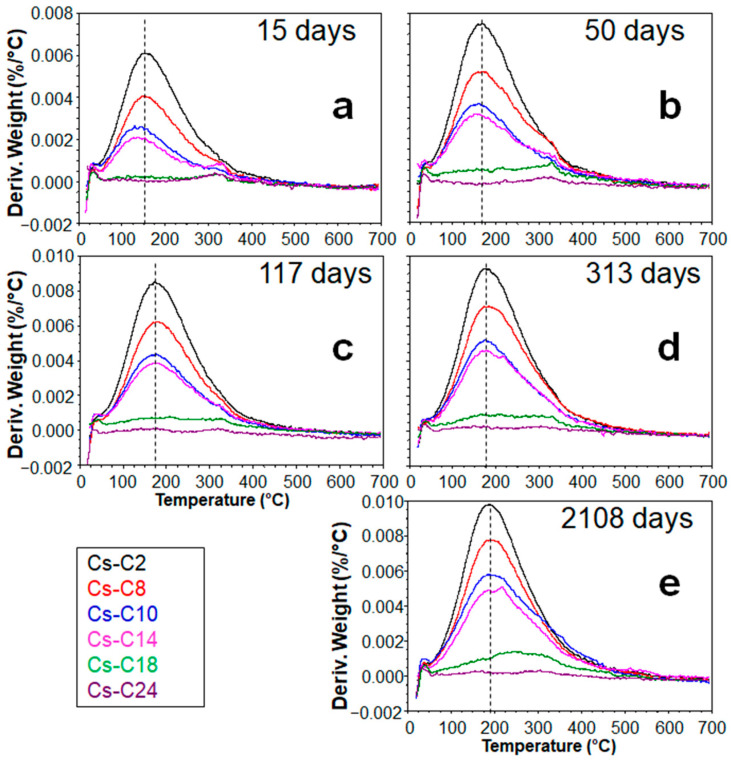
Derivative thermogravimetric (DTG) curves of the materials after (**a**) 15, (**b**) 50, (**c**) 117, (**d**) 313, and (**e**) 2108 days of aging at 21 °C and 55% RH.

**Figure 5:**
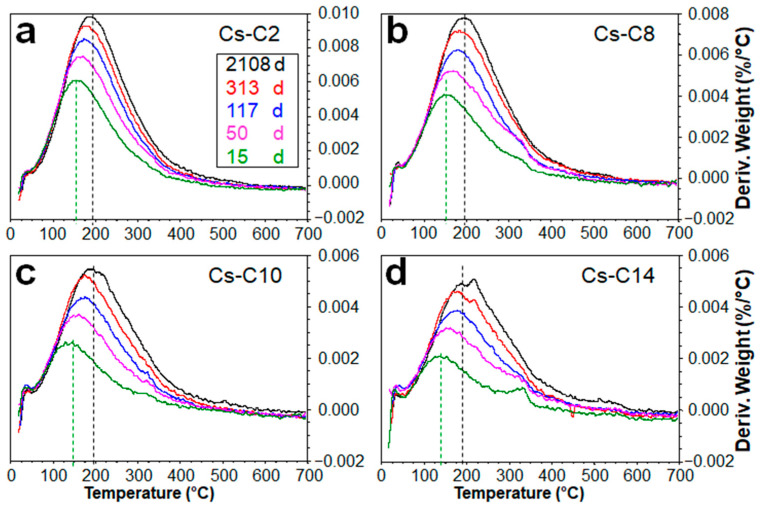
Changes in the DTG peak over time in the samples (**a**) Cs-C2, (**b**) Cs-C8, (**c**) Cs-C10, and (**d**) Cs-C14. The shift of the peak along the temperature axis is shown by the dashed lines.

**Figure 6:**
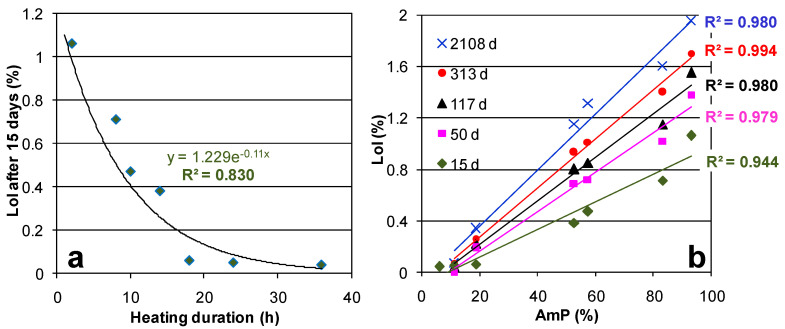
LoI of the samples stored at 21 °C and 55% RH (**a**) after 15 days of aging in relation to the duration of sample heating at 1100 °C and (**b**) after different aging times in relation to the AmP content.

**Figure 7:**
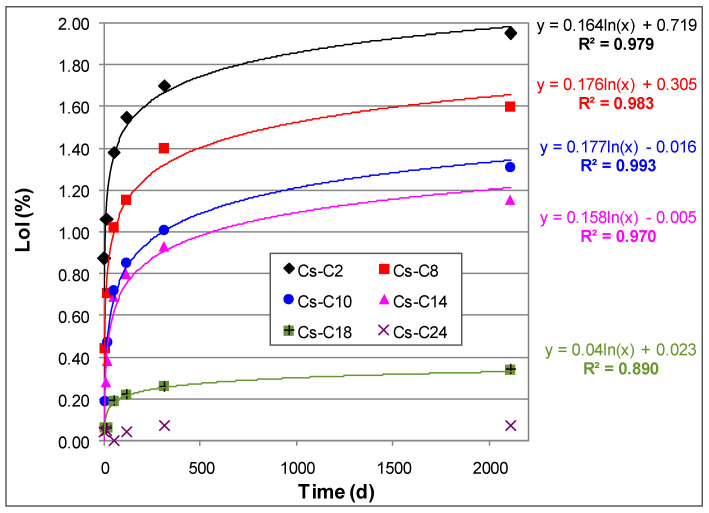
LoI of the samples vs. aging time at 21 °C and 55% RH.

For a given aging time, the temperature of the DTG peak was found to be essentially the same for the four most hydrated samples (Cs-C2, Cs-C8, Cs-C10, and Cs-C14), where the peak intensity and width increased with the amount of H_2_O lost (Figure 4a–e). On the other hand, Figure 5 evidences that, in the same four samples, the DTG peak showed a progressive shift (of ≈45 °C) toward higher temperatures over time. By considering the data at 15 days (which also include Cs-C36), it can be noted that the LoI decreased exponentially with the duration of heating at 1100 °C, as shown in Figure 6a. Moreover, as shown in Figure 6b, the hydration grew linearly with the quantity of the AmP. Furthermore, Figure 6b shows that this direct relationship was maintained over time, with correlation coefficients *R^2^* ranging from 0.944 to 0.994. After almost six years at 21 °C and 55% RH, the water content reached 1.95% in the sample richest in amorphous (Cs-C2), which is a value that is 28 times higher than that of Cs-C24, i.e., the most crystalline material (which essentially showed no weight gain (0.07%, see Table 2)). Figure 7 displays that, except for Cs-C24, the water content increased over time according to a logarithmic trend in all of the samples, and the *R^2^* coefficients varied from 0.890 to 0.993.

The X-ray patterns, which were collected nearly six years after the preparation of the materials, were found to overlap with the initial ones.

### 2.3. Hydration over Time of the Samples Submitted to TG

**Figure 8:**
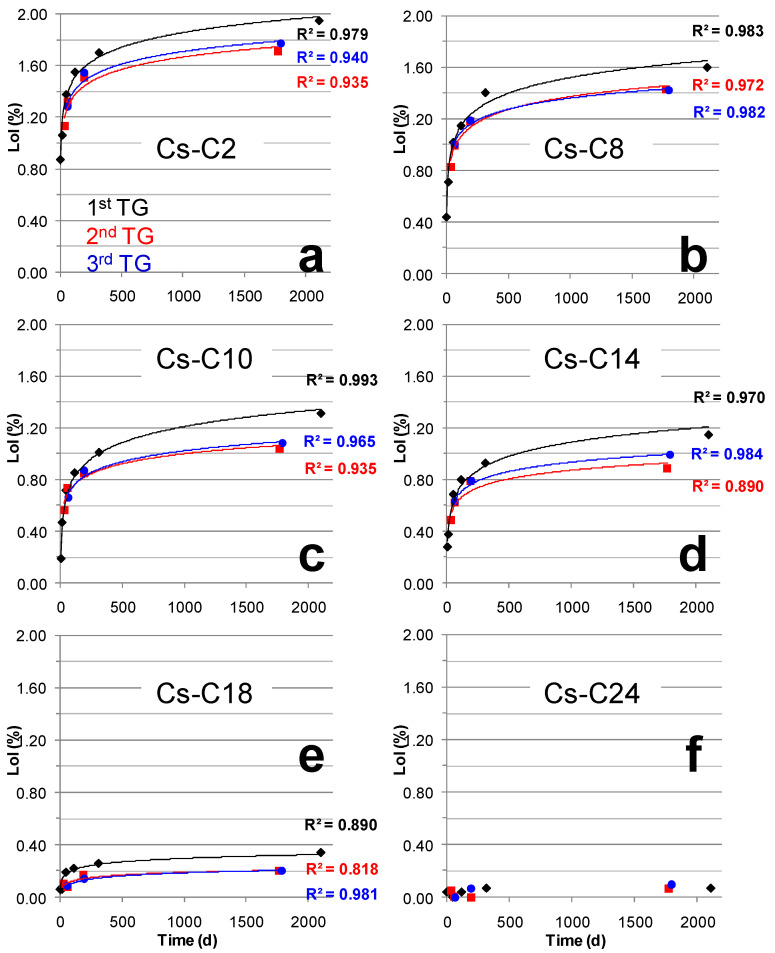
LoI vs. aging time of the materials (which were stored at 21 °C and 55% RH) that were submitted to one, two, and three thermal analyses (see text for explanations). Samples (**a**) Cs-C2, (**b**) Cs-C8, (**c**) Cs-C10, (**d**) Cs-C14, (**e**) Cs-C18, and (**f**) Cs-C24.

Table 3 and Table 4 report the LoI of the samples in relation to the time elapsed since the first and second thermal analyses, respectively. The data show that, after the heating to 700 °C applied during the TG analysis, the materials stored at 21 °C and 55% RH progressively re-hydrated over time. Furthermore, again, the water uptake was found to always be directly related to the amount of AmP in the samples (Figure S2). Figure 8 presents the relationship between the LoI and aging time of the materials subjected to one, two, and three thermal analyses. Once submitted to the first thermal analysis, the samples were no longer able to hydrate following the initial path (see the black lines in Figure 8a–e). In fact, their weight gain over time is shown in the figure by way of lines that run below the initial ones (see the second and third TG in Figure 8a–e). The hydration pathways after two and three TGs were substantially coincident and, for samples from Cs-C2 to Cs-C14, showed LoI values that, after 500 days, were approximately 0.20% lower than those of the first thermal analysis (Figure 8a–d). Such differences decreased to approximately 0.14% for Cs-C18, and they vanished in the most crystalline sample because—essentially—it was never hydrated (Figure 8e–f).

## 3. Discussion

The preparation process allowed one to obtain a series of materials that possessed the same bulk chemistry but different AmP/CAS ratios, with the two phases representing at least 94% of the sample. Storage at room temperature and 55% RH resulted in a different hydration of the materials over time (from ≈0 to almost 2%), and the results showed that hydration increased with the amorphous fraction. Furthermore, the direct relationship between the amount of AmP and the quantity of H_2_O adsorbed was maintained over time (Figure 6b), and this correlation was also observed for the hydrations that followed the TG analyses (Figure S2). According to several authors, glass hydration is absent or negligible when there is an RH < 50% [30,31]; however, the hydration of the AmP, although limited, occurred despite the aging that took place at room temperature and when the humidity was close to the threshold value reported by the literature. By extrapolating from the data in Figure 6b, a completely amorphous sample should reach a water content of 2.1% in six years. The small differences between the specific surface areas of the powdered samples did not influence the kinetics of alteration [39], and the S_BET_ values were not found to be correlated with the observed hydrations. Despite possessing an open framework, CAS does not host water molecules because the size (2.4 × 4.7 Å) of the one-dimensional channels—which run along the *c*-axis—and the presence of Cs^+^ inside them do not allow for the diffusion of H_2_O [40]. Fisch et al. [40] reported that CAS remains anhydrous even after partial Na exchange after 35 days in boiling 1 M NaCl solution. The data presented in this study indicate that, under the tested conditions, CAS does not hydrate, not even after six years of aging. The third component present within the prepared materials was POL, but it may have delivered only a minimal effect on the hydration behavior of the samples since its highest content in the materials is just 5.2% (Cs-C24). However, unlike natural pollucite, which can contain water, POL is anhydrous [40]. Moreover, also in this case, the diameter (~2.86 Å) of the six-membered ring channels parallel to [111], and the cesium inside them, prevented the diffusion of H_2_O [23]. Note that, once dehydrated, natural pollucite also does not take up water from vapor [22]. Overall, it is reasonable to think that POL, just like CAS, did not contribute to the long-term hydrations that were observed in the samples. According to Bunker [41], in silicate glasses, when the ratio between the free ring opening to the kinetic diameter of the water molecule (2.8 Å) is <1, the dominant mechanism for the penetration of water is through hydrolysis, whereas the diffusion of molecular H_2_O is possible when the ratio is >1. Sometimes, the AmP obtained from a thermally treated clinoptilolite still shows a limited re-hydration ability, which is due to the remains of the zeolite channel system. Through this residual open microporosity, water molecules can diffuse; however, in this case, the weight gain—although limited—is fast (e.g., 1.57% in 24 h for amorphized Zn-clinoptilolite kept at room temperature and 50% RH [42]). The LoI values of Cs-C2 and Cs-C8 two days after preparation were 0.87 and 0.44% (Table 2), which corresponded to the 44.6 and 27.5% of the hydration that was reached after almost six years (1.95 and 1.60%, respectively). This meant that the diffusion of the molecular H_2_O may have accounted for only a portion of the hydration that occurred in these samples. Interestingly, Cs-C2 and Cs-C8 had the same density (Figure 2b) but—due to the different duration of the heating at 1100 °C—their AmP fractions should have a different residual *open* microporosity, otherwise their ratio LoI_2days_/LoI_2108days_ would be similar. The importance of the hydration through the penetration of water molecules (as intact species) into the AmP should have been even less in the other samples; in fact, Table 2 shows that the LoI_15days_/LoI_2108days_ ratios decreased progressively from Cs-C2 to Cs-C18 (recall that Cs-C24, essentially, did not hydrate) and that—except in the first sample—most of the hydration occurred from 15 days onward. It is generally recognized that the hydration of glasses kept in atmospheric conditions is mainly due to the hydrolysis of the silicate network, which controls the kinetics of the hydration process [31,32,33,34]. According to Alloteau et al. [32], the process develops in three phases: (a) via adsorption of H_2_O on the glass surface, (b) via penetration of the water molecules into the glass (where there is a dissociation of some of them on the non-bridging oxygen sites according to the acid–base reactions), and (c) via the depolymerization of the network via hydrolysis, which is driven by hydroxide ions. The interdiffusion of water and the hydrolysis of the network can occur simultaneously but at different rates. When the hydration of a glass is controlled only via a diffusion process, the increase in water shows a linear relationship with the square root of time [28,34,43]. This type of relationship stands only for the first three points of the Cs-Cs2, Cs-C8, Cs-C10, and Cs-C14 samples (see the red lines in Figure S3a–d). In reality, the observed hydration rates showed a decrease after approximately 117 days (Figure 7), and the same phenomenon was then recorded regarding the hydrations following the TG analyses (Figure 8). Similar trends have already been reported in the literature, where the time position of the inflexion is affected by the temperature, the RH%, and glass composition [28,29,30,31]. It should be emphasized that, in our experiment, the logarithmic curves showed high correlation coefficients that allow one to describe the hydration paths of all of the samples over time (Figure 8a–e). The trends remained the same, even when the hydration rates were evaluated by considering only the mass loss above 150 °C (Figure S4 and Tables S1–S3), as has been suggested by certain authors when trying to eliminate the contribution provided by weakly bound molecular water [32,34]. The slowdown of glass hydration rates is often attributed to the passivating effect of the altered layer that is formed under unsaturated vapor conditions [28,29,30], which acts as a diffusive barrier for reactive species [30]. It has been reported that, in the hydrated layer, water is present as silanol groups and that molecular H_2_O has strong hydrogen bonds [32,34]. The increase in the fraction of tightly bound water over time explains why, for a given sample, the temperature at which the dehydration ends increases with time (Figure 3). It also explains why the DTG peak shifts progressively toward higher temperatures with aging (Figure 5).

According to the conditions of the tests, alkali depletion and the formation of secondary phases may or may not occur in the alteration layer, whereas an amorphous hydrated layer is always present [28,29,30,31,32,33,34]. The X-ray patterns collected after almost six years of aging of the samples overlapped with the initial ones but, strictly speaking, this may not be sufficient in terms of excluding the possible formation of secondary phases (whose amounts could be below the detecting limit of the technique used). Evidently, the hydrated layer cannot be detected on diffractograms as it is amorphous like the pristine AmP.

The depolymerization of the silicate network in the hydrated layer of altered glasses was demonstrated on the basis of NMR and Raman spectra [30,32,34]. Si enrichment is a recurring feature of the hydrated layer, and it has also been observed in glasses that are aged up to 1 year in a museum-like atmosphere (21–24 °C, 45–75% RH [33]). Due to hydrolysis, the surface of an altered glass contains more silanol groups (Si-OH) when compared to its pristine counterpart; however, in certain cases, a re-polymerization of the network may occur as the silanols can react with each other to form siloxane bonds (Si-O-Si) [28,32,33,34,41]. This is the key to explaining the lower hydration of our samples after the first thermal analysis (Figure 8a–e); in fact, as the hydroxyls were removed during the heating up to 700 °C, the silanol groups disappeared and siloxanes were formed. This phenomenon restructured the AmP network and created an outer film with a degree of polymerization (Q_n_) that was possibly higher when compared to that on the surface of the pristine sample; hence, it was found to be more resistant to hydrolysis (reactivity follows the order Q_1_ > Q_2_ > Q_3_ > Q_4_, where the number refers to the number of bridging oxygen atoms [41]). This explanation agrees with what Warring et al. [44] reported on amorphous silica: “*calcined silica surfaces have greater siloxane relative populations due to the irreversible condensation of silanol groups and are more hydrophobic*”. Note that a further reduction in hydration ability cannot be achieved by repeating the same aging and heating cycle; in fact, the curves of the LoI recorded during the third thermal analysis overlapped with those of the second (Figure 8a–e).

Another aspect that had to be considered in the alteration process of the samples concerned the different Cs_2_O contents in their amorphous fractions (Table 1). The composition of the glass affects the rate of its hydration by vapor, such as—for example—the fact that Al increases resistance to hydrolysis, as well as that the type and quantity of network modifiers also play an important role [29,32,33,34]. It has been hypothesized that the rate of the hydrolysis reaction in atmospheric conditions increases as the hydration energy of the alkaline and alkaline–earth elements contained in the glass decrease, thus helping to explain the lower durability of K_2_O-bearing glasses when compared to soda-lime ones [34]. Based on the hypothesis stated above (which is valid only if the temperature is ≤40 °C [34]), the alteration should be even faster in a glass containing Cs_2_O given the lower hydration enthalpy of Cs^+^ when compared to K^+^. Table 1 shows that, due to the crystallization of CAS and POL, not only does the amount of AmP decrease from Cs-C2 to Cs-C36, but its composition also changes; in fact, the amorphous fraction becomes progressively poorer in Cs_2_O, thus rendering the AmP more durable. This could explain the particularly low final hydration value of Cs-C24 when compared to Cs-C18 (0.07 vs. 0.34%, Table 2), and this is even despite the fact that quantities of the AmP were not that different (11.3 vs. 18.7%, Table 1). The ratio of the LoI_2108_ value to the AmP content of a sample ideally provides the hydration that it would have achieved after six years if it had only consisted of the AmP. As for the samples from Cs-C2 to Cs-C14, the calculated value spanned from 1.92 to 2.28% (average 2.12%, standard deviation 0.15%), whereas it was lower for Cs-C18 (1.82%) and—in particular—Cs-C24 (0.62%), thus confirming the higher durability of the AmP contained in these two samples. In summary, under the tested conditions, the main factor that controlled the hydration of the samples was the amount of the AmP, but its chemical composition may also have played a role (albeit a secondary one). According to the literature, the lower the glass pre-hydration by vapor, the lower the sensitivity to the subsequent leaching should be [25,28,30]. Based on this concept, as well as in taking into account the research results, the amount of the AmP should be minimized as much as possible to achieve maximum cesium immobilization through the heat treatment of Cs-clinoptilolite. In this way, not only will the material be less prone to hydration (and therefore leaching), but the residual AmP will also be poor in Cs because the element will be primarily trapped in the more durable crystalline phases of CAS and POL.

## 4. Materials and Methods

### 4.1. Starting Material

The present research was performed using an aliquot of the Cs-clinoptilolite-rich powder (Cs-C), which was prepared and characterized by Brundu and Cerri [16]. To be brief, the preparation involved the beneficiation of a Sardinian lithotype (the process of which is carefully described in [45]), which resulted in a powder containing 89.5 ± 2.0 wt% of clinoptilolite, as well as minor amounts of volcanic glass, feldspars, opal-CT, quartz, and biotite [16]. To obtain Cs-C, the material was initially conducted in Na-form (11 exchange cycles, 2 h each, at 65 °C in a 1 M NaCl solution), and it was then contacted under continuous stirring with a 0.5 M solution of CsCl at 65 °C (5 cycles, 2 h each). The chemical composition of Cs-C, which was taken from [16], was the following (values in wt%): SiO_2_—55.05; Al_2_O_3_—10.29; Cs_2_O—23.85; Fe_2_O_3_—0.61; MnO—0.01; MgO—0.33; CaO—0.26; Na_2_O—0.13; K_2_O—0.29; TiO_2_—0.16; P_2_O_5_—0.03; and LoI—9.09.

### 4.2. Preparation of the Materials with Different Contents of AmP and CAS

Seven aliquots of Cs-C were subjected to thermal treatments at 1100 °C for different durations (2, 8, 10, 14, 18, 24, and 36 h, respectively). The powders were then placed in high alumina crucibles (Coors™, Merk Life Sciences, Milano, Italy), which were heated in a muffle furnace (Vittadini mod. FS.3, Controls, Milano, Italy) and then finally cooled in air. The materials were labeled as follows: Cs-C2, Cs-C8, Cs-C10, Cs-C14, Cs-C18, Cs-C24, and Cs-C36. After heating, the samples were micronized (with a Retsch MM400 mill that was equipped with ZrO_2_ jars and balls) not only to obtain a particle size that was suitable for quantitative X-ray analyses (1–5 μm [46]), but also to minimize the possible differences in the specific surface of the materials, thereby preventing this aspect from affecting the results in terms of hydration. Since, at room temperature and 55% RH, hydration is limited in the foreseeable, an increase in the specific surface area of the materials should boost the kinetics of the hydration process, thus making it easier to detect any differences in the hydration behavior of the samples. For this reason, the powders were not pressed to prepare solid forms such as the disks that are sometimes used to test the physical and chemical properties of materials aimed at immobilizing Cs [17,47,48]. After preparation, a fraction of each sample was stored under controlled conditions of the temperature and RH% (see Section 4.5), which was performed to evaluate its hydration over time, while the rest was used for the other analyses.

### 4.3. XRD Analyses

The mineralogical composition of the materials was investigated by XRD using a Bruker D2-Phaser equipped with a LynxEye PSD detector and employing low-background Si-crystal sample holders (Bruker, Karlsruhe, Germany). The experimental parameters were applied as follows: 30 kV, 10 mA, CuKα radiation, 2θ range 6–70°, a step size of 0.020°, the detector opening at 5°, and the spinner at 15 rpm. The materials were subjected to an initial qualitative analysis that was performed by setting a time per step of 0.5 s and then identifying the mineral phases on the X-ray patterns with the software EVA 14.2 (Bruker DIFFRAC plus Package), which was coupled with the database PDF-2 (International Centre for Diffraction Data, Philadelphia, PA, USA). At the end of the study, qualitative analyses were also carried out on the samples that were kept for nearly six years under controlled conditions. For the quantitative analysis, the samples were preliminarily mixed with corundum, which was used as the internal standard (20 wt% of α-Al_2_O_3_, 1 μm, Buehler micropolish II), and the measurements were then collected by setting a time step of 2 s. The amounts of the crystalline phases and of the amorphous component were determined by the Rietveld method employing Bruker Topas 5 software (the crystal structures and atomic coordinates of minerals were obtained from the International Crystal Structure Database [49]).

### 4.4. Density and Specific Surface Area Analyses

The density of the materials was determined using a helium pycnometer Micromeritics AccuPyc 1340, performing five measurements for each sample. The specific surface area (S_BET_)—calculated according to the Brunauer–Emmett–Teller method—was assessed via gas adsorption using a Micromeritics Flowsorb II 2300.

### 4.5. Storage at Controlled Temperature and RH%

The samples were placed in a desiccator that contained a saturated solution of calcium nitrate on the bottom (Sigma-Aldrich, Milano, Italy chemicals, purity ≥ 99%). A temperature and humidity data logger (EBRO EBI-TH1) was placed inside the container next to the samples. This protocol coincided with that which was adopted by other authors to study the vapor hydration of glass [25,31]. Following a procedure that was already used successfully [50], the desiccator remained in a room in the basement, which was equipped with an autonomous climatization system for the entire duration of the study. This helped to limit the temperature and RH fluctuations in the desiccator to 21.0 ± 3.5 °C and 55 ± 5%, respectively. Small amounts of the saline solution were added over the years to keep the volume constant within the desiccator.

### 4.6. TG-DTG Analyses

The hydration caused, over time, an increase in the weight of the materials. This was evaluated through TG and DTG analyses, wherein the mass of water that was lost during heating via a TA Instrument Q600 (Centro Servizi di Ateneo per la Ricerca, CeSAR—University of Sassari) was measured. Aliquots of approximately 20 mg of the powders stored under controlled conditions (see Section 4.5) were put in an alumina crucible and heated to 700 °C at a rate of 10 °C/min in static air. To investigate the hydration over time, different aliquots of the samples were analyzed after a storage time of the following:

(a) 1 day (Cs-C18 and Cs-C24), 2 days (Cs-C2 and Cs-C8), 4 days (Cs-C10), and 8 days (Cs-C14);
(b) 15 days (all samples);
(c) 50 days (all samples except Cs-C36);
(d) 117 days (all samples except Cs-C36);
(e) 313 days (all samples except Cs-C36);
(f) 2108 days (all samples except Cs-C36).

Due to vial damage, Cs-C36 was lost during laboratory operations, which is why only one analysis was performed on it. Once analyzed, the aliquots referred to in points (b) to (e) were recovered and stored again in the desiccator at a controlled temperature and RH%.

To evaluate the hydration path over time of the materials subjected to one thermal analysis, a schema was applied as follows:

(g) The aliquots already analyzed in point (b), except Cs-C36, were re-tested after 33 days;
(h) The aliquots already analyzed in point (c) were re-tested after 62 days;
(i) The aliquots already analyzed in point (d) were re-tested after 192 days;
(l) The aliquots already analyzed in point (e) were re-tested after 1772 days.

Again, once analyzed, the powders referred to in points (g) to (i) were retrieved and put into the desiccator. The hydration of the materials that underwent two thermal analyses was evaluated in accordance with the following schema:

(m) The aliquots already analyzed in point (g) were re-tested after 63 days;
(n) The aliquots already analyzed in point (h) were re-tested after 194 days;
(o) The aliquots already analyzed in point (i) were re-tested after 1796 days.

The results of the thermal analyses were evaluated using TA-Universal Analysis 2000 V 4.5A software.

## 5. Conclusions

Using Cs-clinoptilolite, a set of samples mostly composed of AmP and CAS (the sum of which were ≥94 wt%) and subordinate POL were prepared via a thermal treatment (2–36 h) at 1100 °C. Six samples with an AmP/CsAlSi_5_O_12_ ratio from 26.5 to 0.1 were kept at 21 °C and 55% relative humidity. Their hydration was measured via thermogravimetry (TG) over a period of almost six years. The hydration that resulted was directly related to the AmP quantity. Over time, the water content increased according to a logarithmic trend, and it reached 1.95% in the Amp-richest sample, whereas it attained only 0.07% in the most crystalline material. The hydrolysis of the AmP led to an increase over time in the tightly bound water, which was evidenced—along the DTG curve—by the progressive shift toward higher temperatures (≈45 °C in 6 years) of the dehydration peak. Samples with AmP ≤ 19% resulted in being slightly less prone to hydration due to the lower Cs content in their amorphous component, as well as due to their cesium being preferentially incorporated into CAS and POL. During the TG analysis, the dehydroxylation of the silanol groups led to a re-polymerization of the amorphous network, and this occurred because silanols react with each other to form siloxanes. This should explain why the hydration paths of the samples subjected to TG do not trace the initial one but instead follow a parallel curve slightly below it.

## Figures and Tables

**Table 1:** Mineralogical compositions (wt%) of the materials, the Cs-occupancy for CAS, the cesium content in the phases referred to the total in the sample, and the calculated amount of Cs_2_O in the amorphous phase (AmP).

Sample	CAS	POL	Feld. *	AmP	AmP + CAS	AmP/CAS	Cs-Occup.	Cs Phase/Cs Sample (%)			Cs_2_O%
							in CAS	CAS	POL	AmP	in AmP
Cs-C2	3.5	0.4	3.2	92.9	96.4	26.5	1.00	3.8	0.7	95.5	26.9
e.s.d. **	*±0.8*	*±0.1*	*±0.6*	*±3.0*							
Cs-C8	15.4	1.4	-	83.2	98.6	5.4	0.92	15.9	2.4	81.7	25.7
*e.s.d.*	*±2.6*	*±0.2*		*±2.8*							
Cs-C10	39.1	3.5	-	57.4	96.5	1.5	0.93	40.6	6.0	53.4	24.4
*e.s.d.*	*±3.5*	*±0.5*		*±2.5*							
Cs-C14	43.1	4.2	-	52.7	95.8	1.2	0.93	44.7	7.2	48.1	23.9
*e.s.d.*	*±3.8.*	*±0.7*		*±2.3*							
Cs-C18	76.3	5.0		18.7	95.0	0.2	0.92	78.1	8.6	13.3	18.7
*e.s.d.*	*±4.2*	*±0.8*		*±2.0*							
Cs-C24	83.5	5.2	-	11.3	94.8	0.1	0.91	85.0	9.0	6.0	14.0
*e.s.d.*	*±4.4*	*±0.8*		*±1.6*							
Cs-C36	87.7	6.1	-	6.2	93.9	0.1	0.91	89.6	10.4	0	0
*e.s.d.*	*±4.7*	*±1.1*		*±1.4*							

* Feldspar; ** estimated standard deviations.

**Table 2:** Loss on ignition (LoI) in wt% of the materials, which were stored at 21 °C and 55% relative humidity (RH), referred to the time elapsed since their preparation. The error on the LoI measurements is ≤±0.03%.

Time (Days)	Cs-C2	Cs-C8	Cs-C10	Cs-C14	Cs-C18	Cs-C24	Cs-C36
1	-	-	-	-	0.06	0.04	-
2	0.87	0.44	-	-	-	-	-
4	-	-	0.19	-	-	-	-
8	-	-	-	0.28	-	-	-
15	1.06	0.71	0.47	0.38	0.06	0.05	0.04
50	1.38	1.02	0.72	0.69	0.19	0.00	-
117	1.55	1.15	0.85	0.80	0.22	0.04	-
313	1.70	1.40	1.01	0.93	0.26	0.07	-
2108	1.95	1.60	1.31	1.15	0.34	0.07	-
LoI_15d_/LoI_2108d_	0.54	0.44	0.36	0.33	0.18	0.71	-

**Table 3:** LoI (wt%) of the materials, which were stored at 21 °C and 55% RH, in relation to the time elapsed after their first thermal analysis. The error on the LoI measurements is ≤±0.03%.

Time (Days)	Cs-C2	Cs-C8	Cs-C10	Cs-C14	Cs-C18	Cs-C24
33	1.13	0.83	0.56	0.49	0.10	0.05
62	1.32	0.99	0.74	0.62	0.08	0.01
192	1.51	1.18	0.85	0.79	0.17	0.00
1772	1.71	1.43	1.04	0.89	0.20	0.07

**Table 4:** LoI (wt%) of the materials, which were stored at 21 °C and 55% RH, in relation to the time elapsed after their second thermal analysis. The error on the LoI measurements is ≤±0.03%.

Time (Days)	Cs-C2	Cs-C8	Cs-C10	Cs-C14	Cs-C18	Cs-C24
63	1.28	1.00	0.66	0.63	0.09	0.00
194	1.55	1.19	0.87	0.79	0.14	0.07
1796	1.77	1.42	1.08	0.99	0.20	0.10

## Data Availability

The data supporting the findings are available within the article and in the Supplementary Materials.

## Supplementary Materials

The following supporting information can be downloaded at: https://www.mdpi.com/article/10.3390/molecules29061302/s1, Figure S1: screenshots of the refinements (software Bruker Topas 5—Rietveld method) showing the comparison between the observed and the calculated XRD pattern for (a) Cs-C2, (b) Cs-C8, (c) Cs-C10, (d) Cs-C14, (e) Cs-C18, (f) Cs-C24, and (g) Cs-C36; Figure S2: relationship between loss on ignition (LoI) and content of amorphous phase (AmP), referred to elapsed time at 21 °C and 55% relative humidity (RH) since their (a) first and (b) second thermal analysis; Figure S3: LoI of the samples vs. square root of aging time; Figure S4: LoI above 150 °C vs. aging time at 21 °C and 55% RH of the materials submitted to one, two, and three thermal analyses; Table S1: LoI (wt%) above 150 °C of the samples stored at 21 °C and 55% RH, referred to the time elapsed since their preparation; Table S2: LoI (wt%) above 150 °C of the samples stored at 21 °C and 55% RH, referred to the time elapsed since their first thermal analysis; Table S3: LoI (wt%) above 150 °C of the samples stored at 21 °C and 55% RH, referred to the time elapsed since their second thermal analysis.
