# Supplementary material for: A Six-Year Hydration Evaluation of Cs-Bearing Materials at Room Temperature and 55% Relative Humidity Simulating Radioactive Waste with Different Crystallinities
Source: Molecules. 2024 Mar 14;29(6):1302. doi: 10.3390/molecules29061302 (PMC10975553; doi:10.3390/molecules29061302)
Supplement: Supplementary file 1 [file molecules-29-01302-s001.zip › molecules-2873956-supplementary.pdf]

Supplementary Materials for

A Six-Year Hydration Evaluation of Cs-Bearing Materials at Room Temperature and 55% Relative Humidity Simulating Radioactive Waste with Different Crystallinities

Guido Cerri * and Antonio Brundu

Department of Architecture, Design and Urban Planning—GeoMaterials Lab, Sassari University, Via Piandanna 4, 07100 Sassari, Italy
* Correspondence: gcerri@uniss.it

Figure S1. Screenshots of the refinements (software Bruker Topas 5 – Rietveld method) showing the comparison between the observed (blue) and the calculated (red) XRD pattern for: **a**) Cs-C2; **b**) Cs-C8; **c**) Cs-C10; **d**) Cs-C14; **e**) Cs-C18; **f**) Cs-C24; **g**) Cs-C36. The grey curve is the difference between the observed and the calculated intensity. Corundum has been added as internal standard to quantify the amorphous fraction, and the correct amounts of the phases are reported in the Table 1 of the article. Rwp: reliability factor of the Rietveld refinement.

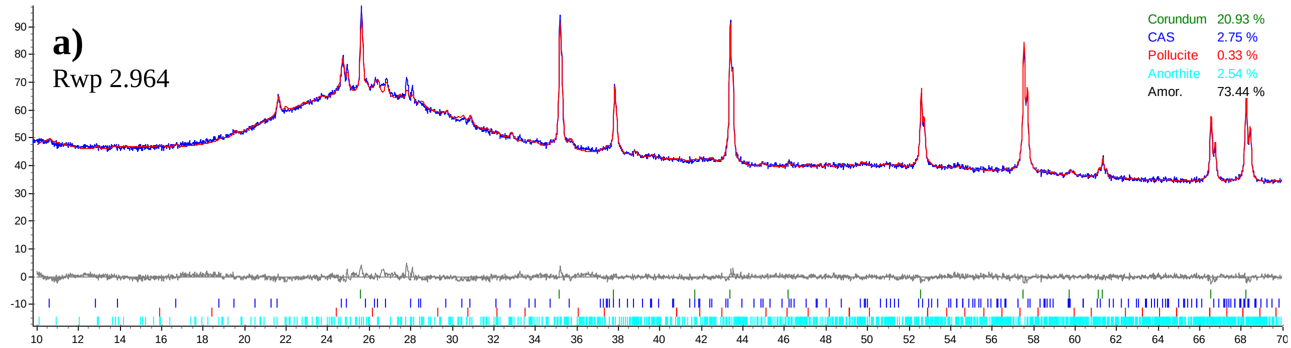

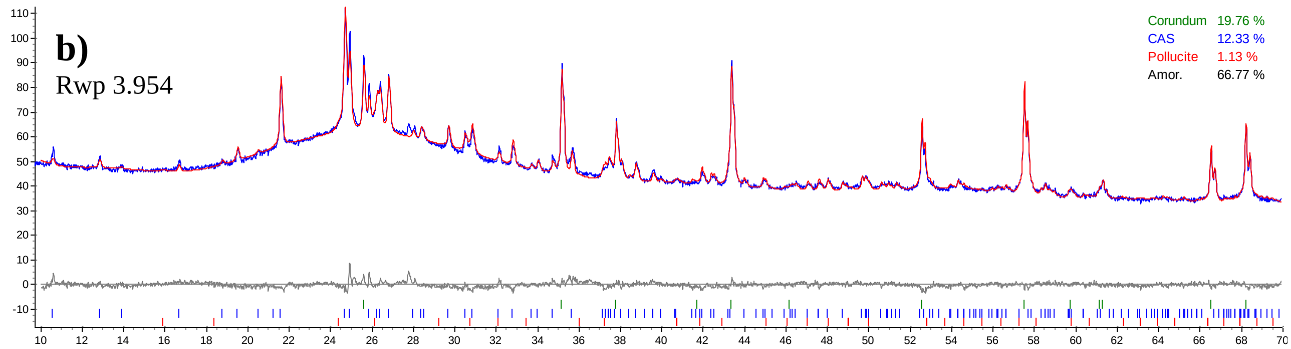

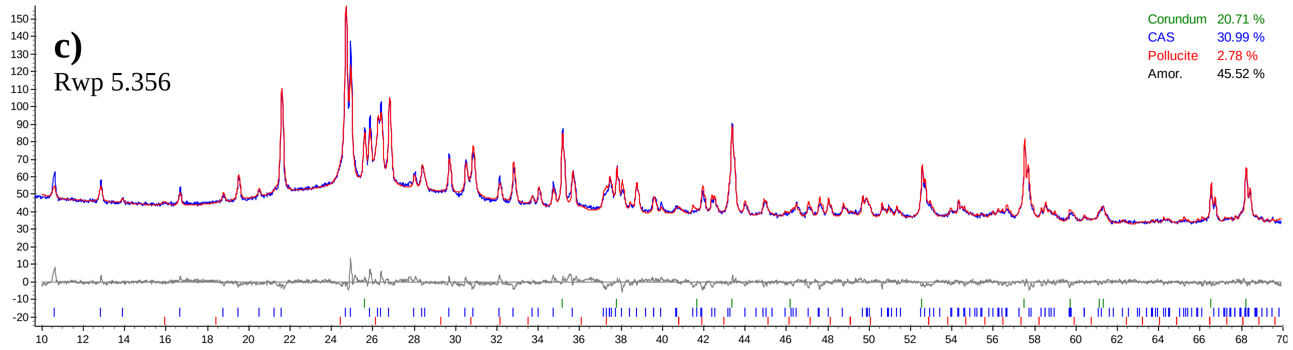
c)
Rwp 5.356
Corundum 20.71 %
CAS 30.99 %
Pollucite 2.78 %
Amor. 45.52 %

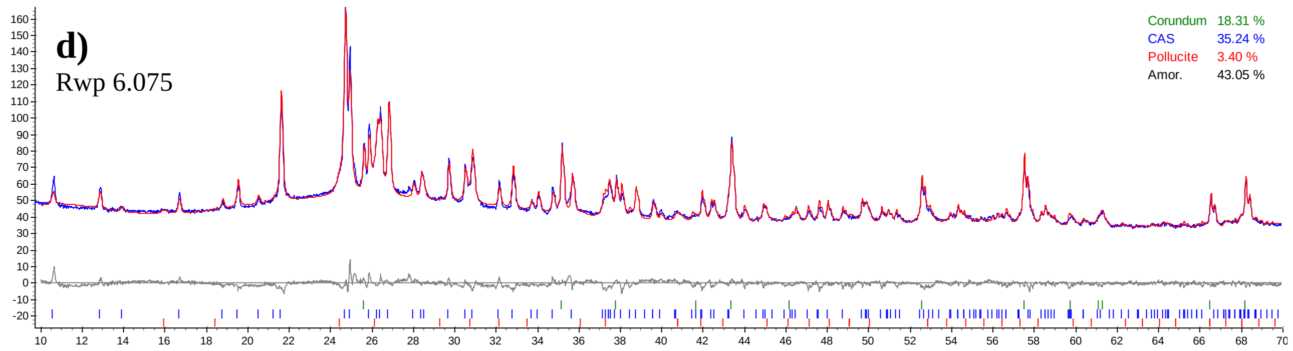
d)
Rwp 6.075
Corundum 18.31 %
CAS 35.24 %
Pollucite 3.40 %
Amor. 43.05 %

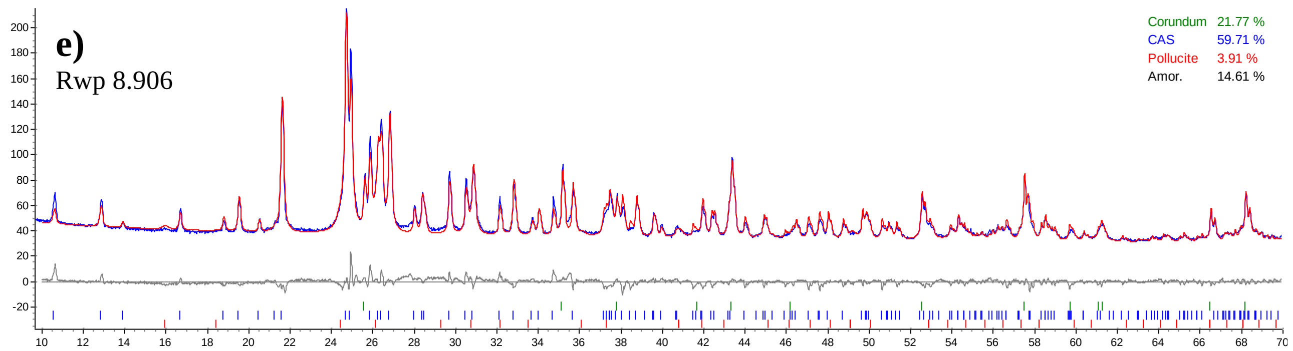
e)
Rwp 8.906
Corundum 21.77 %
CAS 59.71 %
Pollucite 3.91 %
Amor. 14.61 %

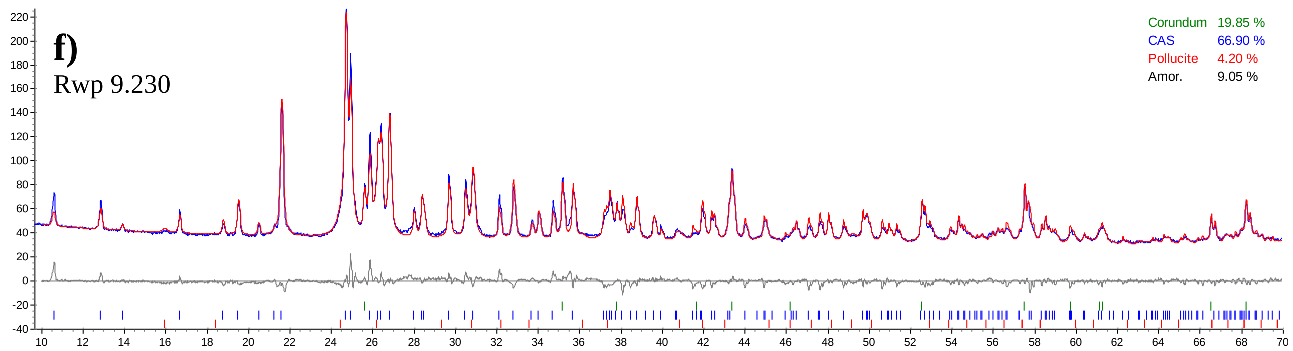
f)
Rwp 9.230
Corundum 19.85 %
CAS 66.90 %
Pollucite 4.20 %
Amor. 9.05 %

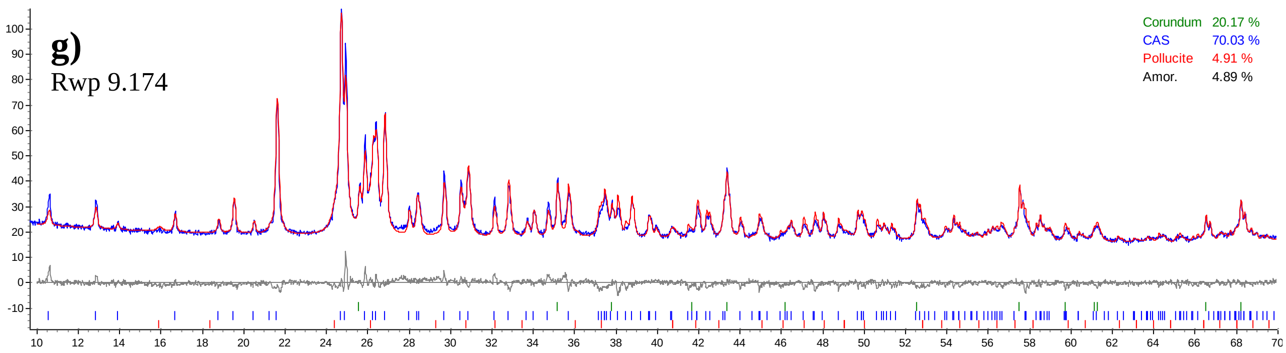

g)
Rwp 9.174
Corundum 20.17 %
CAS 70.03 %
Pollucite 4.91 %
Amor. 4.89 %

Figure S2. Relationship between loss on ignition (LoI) and content of amorphous phase (AmP) referred to elapsed time at 21 °C and 55% relative humidity (RH) since their (**a**) 1st and (**b**) 2nd thermal analysis.

(a)

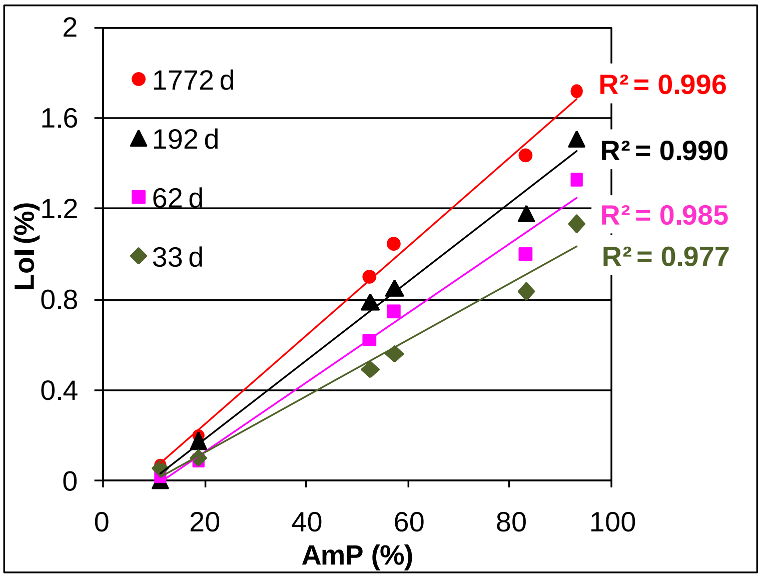

(b)

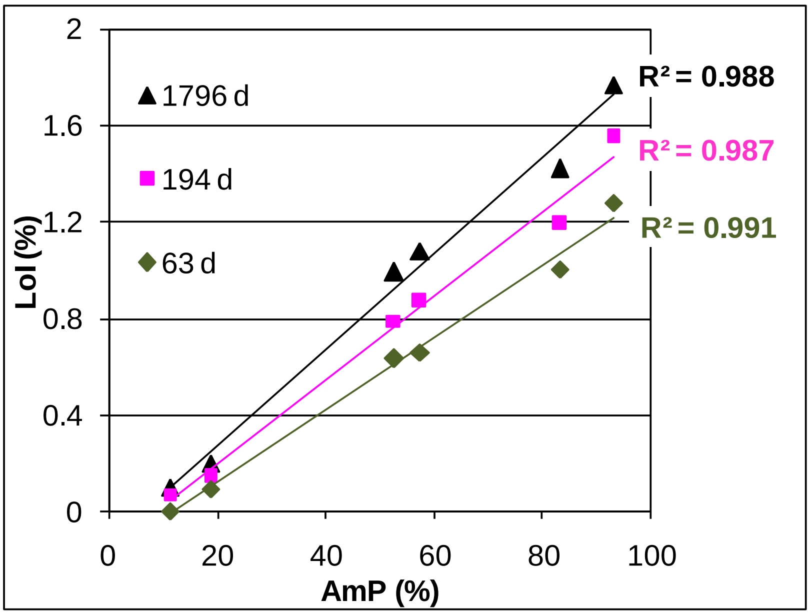

Figure S3. LoI of the samples vs. square root of aging time.

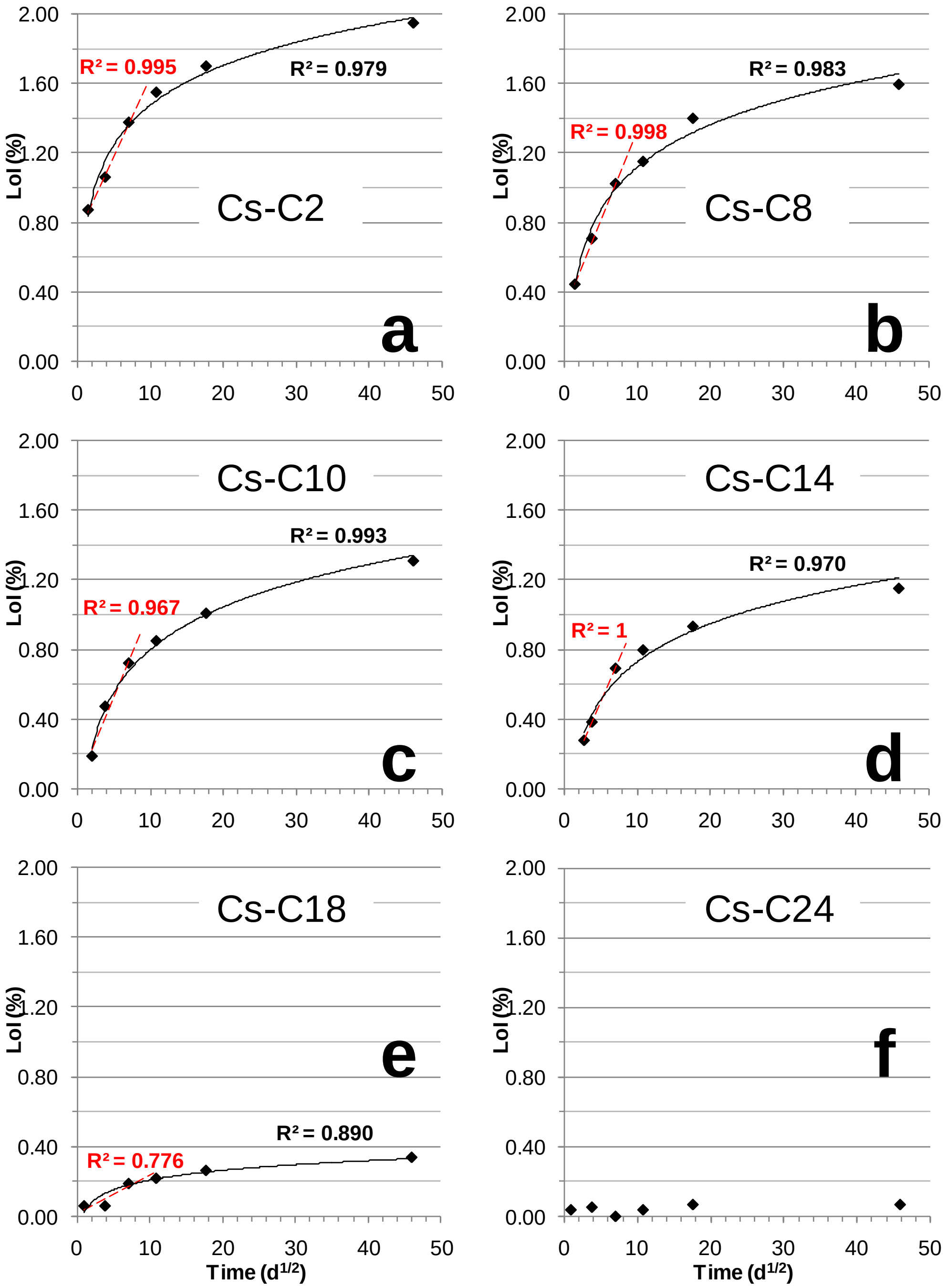

Figure S4. LoI above 150 °C vs. aging time at 21 °C and 55% RH of the materials submitted to one (black lines), two (red lines) and three (blue lines) thermal analyses.

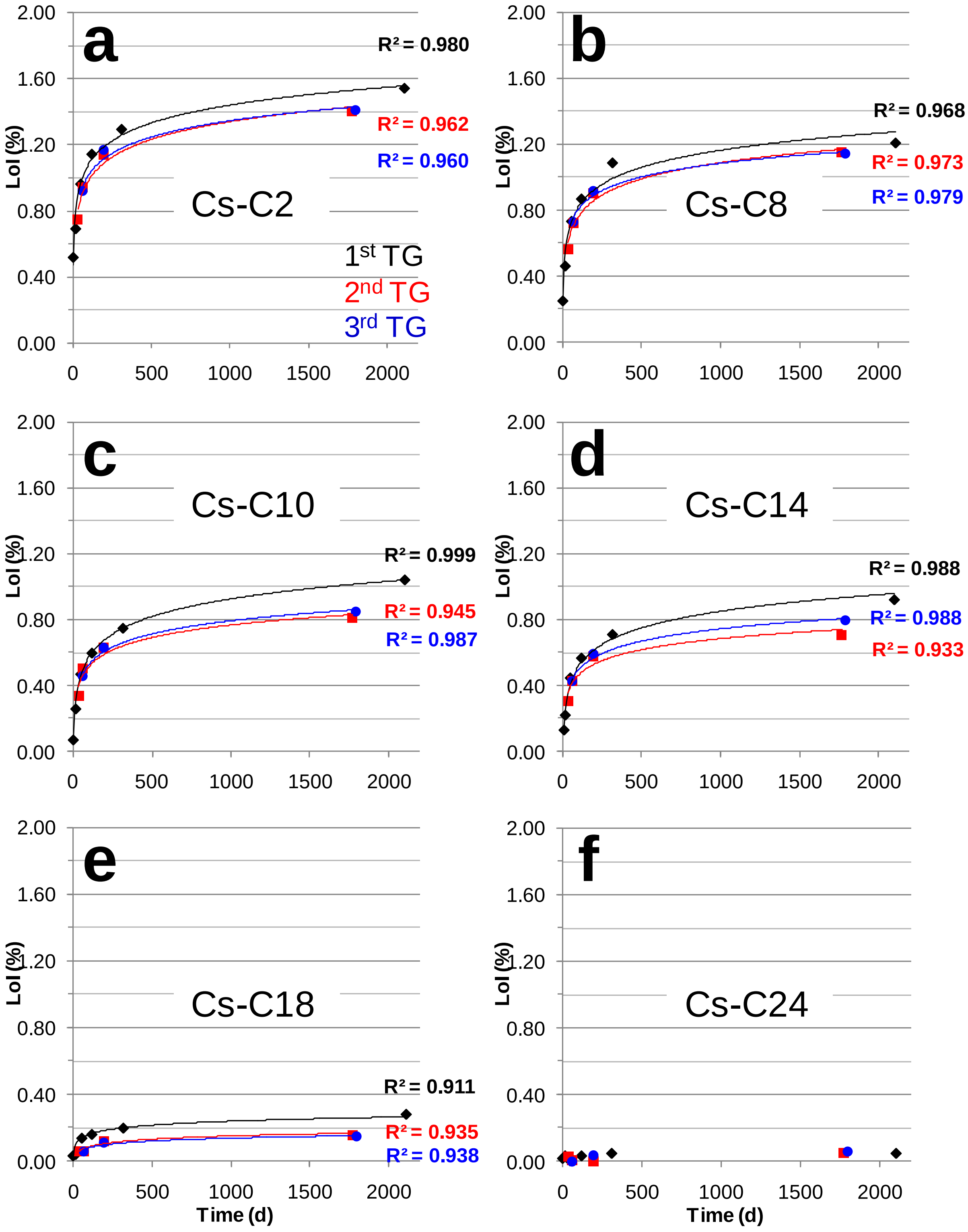

Table S1. LoI (wt%) above 150 °C of the samples stored at 21 °C and 55% RH, referred to the time elapsed since their preparation. The error on LoI measurements is ≤ ±0.03%.

**Time (days)**	**Cs-C2**	**Cs-C8**	**Cs-C10**	**Cs-C14**	**Cs-C18**	**Cs-C24**
1	-	-	-	-	0.03	0.02
2	0.52	0.25	-	-	-	-
4	-	-	0.07	-	-	-
8	-	-	-	0.13	-	-
15	0.69	0.46	0.26	0.22	0.04	0.03
50	0.96	0.73	0.47	0.45	0.14	0.00
117	1.14	0.87	0.60	0.57	0.16	0.03
313	1.29	1.09	0.75	0.71	0.20	0.05
2108	1.54	1.21	1.04	0.92	0.28	0.05

Table S2. LoI (wt%) above 150 °C of the samples stored at 21 °C and 55% RH, referred to the time elapsed since their 1st thermal analysis. The error on LoI measurements is ≤ ±0.03%.

**Time (days)**	**Cs-C2**	**Cs-C8**	**Cs-C10**	**Cs-C14**	**Cs-C18**	**Cs-C24**
33	0.75	0.56	0.34	0.31	0.06	0.03
62	0.94	0.72	0.50	0.43	0.06	0.01
192	1.14	0.90	0.63	0.58	0.12	0.00
1772	1.40	1.15	0.81	0.71	0.16	0.05

Table S3. LoI (wt%) above 150 °C of the samples stored at 21 °C and 55% RH, referred to the time elapsed since their 2nd thermal analysis. The error on LoI measurements is ≤ ±0.03%.

**Time (days)**	**Cs-C2**	**Cs-C8**	**Cs-C10**	**Cs-C14**	**Cs-C18**	**Cs-C24**
63	0.92	0.73	0.46	0.43	0.06	0.00
194	1.17	0.92	0.63	0.59	0.11	0.04
1796	1.41	1.14	0.85	0.80	0.15	0.06
